# Predicting morbidity and mortality in acute pancreatitis in an Indian population: a comparative study of the BISAP score, Ranson’s score and CT severity index

**DOI:** 10.1093/gastro/gov009

**Published:** 2015-03-02

**Authors:** Jitin Yadav, Sanjay Kumar Yadav, Satish Kumar, Ranjan George Baxla, Dipendra Kumar Sinha, Pankaj Bodra, Ram Chandra Besra, Babu Mani Baski, Om Prakash, Abhinav Anand

**Affiliations:** Department of General Surgery, Rajendra Institute of Medical Sciences, Ranchi, Jharkhand, India

**Keywords:** acute pancreatitis, mortality, bedside index for severity in acute pancreatitis (BISAP), Ranson’s score, computed tomography severity index (CTSI)

## Abstract

**Objective:** Our aim was to prospectively evaluate the accuracy of the bedside index for severity in acute pancreatitis (BISAP) score in predicting mortality, as well as intermediate markers of severity, in a tertiary care centre in east central India, which caters mostly for an economically underprivileged population.

**Methods:** A total of 119 consecutive cases with acute pancreatitis were admitted to our institution between November 2012 and October 2014. BISAP scores were calculated for all cases, within 24 hours of presentation. Ranson’s score and computed tomography severity index (CTSI) were also established. The respective abilities of the three scoring systems to predict mortality was evaluated using trend and discrimination analysis. The optimal cut-off score for mortality from the receiver operating characteristics (ROC) curve was used to evaluate the development of persistent organ failure and pancreatic necrosis (PNec).

**Results:** Of the 119 cases, 42 (35.2%) developed organ failure and were classified as severe acute pancreatitis (SAP), 47 (39.5%) developed PNec, and 12 (10.1%) died. The area under the curve (AUC) results for BISAP score in predicting SAP, PNec, and mortality were 0.962, 0.934 and 0.846, respectively. Ranson’s score showed a slightly lower accuracy for predicting SAP (AUC 0.956) and mortality (AUC 0.841). CTSI was the most accurate in predicting PNec, with an AUC of 0.958. The sensitivity and specificity of BISAP score, with a cut-off of ≥3 in predicting mortality, were 100% and 69.2%, respectively.

**Conclusions:** The BISAP score represents a simple way of identifying, within 24 hours of presentation, patients at greater risk of dying and the development of intermediate markers of severity. This risk stratification method can be utilized to improve clinical care and facilitate enrolment in clinical trials.

## Introduction

Several prognostic scoring systems with clinical, biochemical and radiological criteria have been proposed to classify the severity of pancreatitis. The Ranson’s scoring system uses a set of 11 prognostic signs, where three or more criteria indicate the presence of severe acute pancreatitis (SAP). In 2008 Wu *et al.* proposed a new prognostic scoring system for the early determination of the severity of acute pancreatitis, which they named the ‘bedside index of severity in acute pancreatitis’ (BISAP) [1, 2]. BISAP considers five parameters: blood urea nitrogen >25 mg/dL, impaired mental status, systemic inflammatory response syndrome (SIRS), age >60 years, and detection of pleural effusion by imaging.

There have been several studies in developed countries that compared the BISAP score system with (i) Ranson’s, (ii) acute physiology and chronic health evaluation II (APACHE-II) and (iii) computed tomography severity index (CTSI) scores. BISAP has been reported by Wu *et al.* and Papachristou *et al.* to be an accurate tool for risk stratification of acute pancreatitis in western populations [1, 3]. Kim *et al.* concluded that BISAP is more accurate in predicting the severity of acute pancreatitis than the serum procalcitonin (PCT), APACHE-II, Glasgow, and CTSI scores in a Korean population [4]. Zhang *et al.* also reported that the BISAP score may be a valuable means of risk stratification and prognostic prediction in Chinese patients with acute pancreatitis [5]. The present study aimed to evaluate the value of BISAP scoring in an Indian setting, through comparing it with Ranson’s and the CTSI scoring systems.

## Patients and methods

This was a prospective, descriptive study of patients admitted with acute pancreatitis at the Rajendra Institute of Medical sciences (RIMS) from November 2012 to October 2014. The BISAP, Ranson’s and CTSI scores were evaluated in a study population of 119 consecutive cases of acute pancreatitis.

The diagnosis of acute pancreatitis was based on the presence of two of the following three features: (i) characteristic abdominal pain of acute pancreatitis, (ii) serum amylase and/or lipase ≥3 times the upper limit of normal, and (iii) characteristic findings of acute pancreatitis on abdominal CT scan. Patients without next of kin to consent to the study were excluded from the study.

The BISAP score was calculated using data from the 24 hours following admission, while the Ranson’s score used data from the first 48 hours. CTSI was calculated in patients after contrast-enhanced computed tomography (CECT) carried out at any time in the first 7 days of hospitalization. The BISAP and Ranson’s scores were routinely obtained by the surgeon on duty or resident surgical officer at our institution at the time of triage in the emergency room and/or when the patient underwent an initial assessment in the general medical wards or intensive care unit. CTSI data was obtained from radiologists.

Systemic inflammatory response syndrome was defined as two or more of the following: temperature of <36°C or >38°C, PaCO_2_ <32 mmHg or respiratory rate >20 breaths/min, pulse >90 beats/min, and white blood cell count <4000 or >12 000 cells/mm^3^ or >10% immature bands. The Marshall score was used for organ failure. Organ failure scores were calculated for all patients during the first 72 hours of hospitalization, based on the most extreme laboratory value or clinical measurement during each 24-hour period. Duration of organ failure was defined as transient (≤48 h from the time of presentation) or persistent (>48 h). The presence of pleural effusions was determined by a CT scan, chest radiograph, or abdominal ultrasound obtained within 24 hours of presentation. Severe AP was defined as persistent organ failure for more than 48 hours; organ failure included shock (systolic blood pressure <90 mmHg), pulmonary insufficiency (arterial PaO_2_ <60 mmHg with unassisted breathing or the need for mechanical ventilation), or renal failure (serum creatinine level >2 mg/dL after rehydration or haemodialysis).

## Statistical analysis

All the statistical analyses were performed using ‘statistical package for social sciences’ (SPSS) software (SPSS Inc., Chicago, Illinois, USA). Continuous data is presented as mean ± standard deviation (SD). Categorical values were evaluated using χ^2^ or Fisher’s exact test. Trends were evaluated using the Cochran-Armitage test. The receiver operating characteristic (ROC) curve was examined for an optimal BISAP score for mortality prediction. Discrimination of the BISAP score for predicting mortality was evaluated in the prospective cohort, using the area under the ROC curve (AUC). Sensitivity, specificity, positive predictive value (PPV), and negative predictive value (NPV) were calculated for individual scoring systems. A *P-*value of <0.05 was considered to be significant.

## Results

### Patient demographics and complications

A total of 119 consecutive patients with acute pancreatitis were included in this study. Patients' mean age was 38.94 ± 14.59 years. Males were in the majority at 70.6%. Mean body mass index was 29.20 ± 3.21 kg/m^2^. At 40.3%, alcohol was the most common cause of acute pancreatitis in our study, followed by gall stone in 31.1% of cases. For 76 patients (63.9%), this was their first attack of acute pancreatitis. Ninety-eight patients (82.4%) were referred from various hospitals. We observed a mortality of 10.1% (*n = *12) in our study. Complications observed are set out in Table 1.

**Table 1.** Complications

Complications	*n* (%)
Transient organ failure	28 (23.5)
Persistent organ failure	12 (10.1)
Severe acute pancreatitis	42 (35.2)
Pancreatic necrosis	47 (39.5)
Pseudocyst	33 (27.7)

### BISAP score

The proportions of subjects with SAP, pancreatic necrosis (PNec) and mortality, stratified by the BISAP score, are presented in Table 2.

**Table 2.** BISAP score

BISAP score	No. of patients (*n = *119)	SAP (*n = *42)	PNec (*n = *47)	Mortality (*n = *12)
0	13	0	01	0
1	11	0	0	0
2	49	2	5	0
3	23	20	20	0
4	18	15	16	7
5	5	5	5	5

BISAP = bedside index for severity in acute pancreatitis; PNec = pancreatic necrosis; SAP = severe acute pancreatitis

Cochran-Armitage trend test *P-*values for SAP, PNec, and mortality were <0.001, <0.001, and <0.01, respectively.

### Comparison of scoring systems in predicting SAP, PNec, and mortality

**Figure 1.**
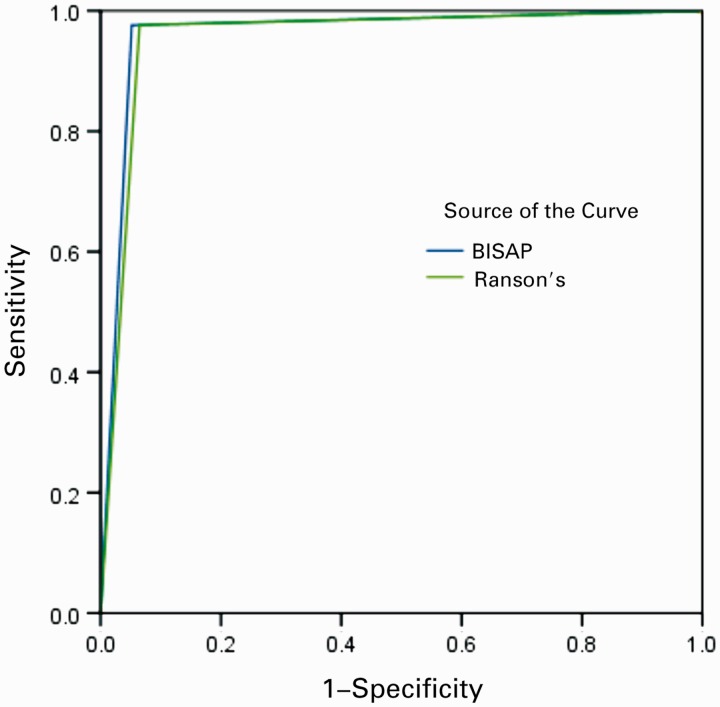
ROC curve for severe acute pancreatitis. BISAP = bedside index for severity in acute pancreatitis; CTSI = computed tomography severity index.

**Figure 2.**
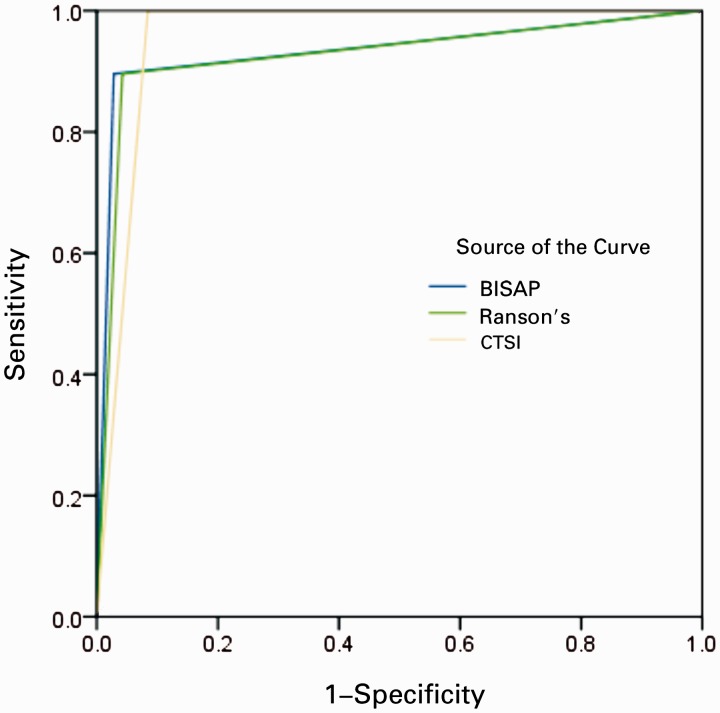
ROC curve for necrotizing pancreatitis. BISAP = bedside index for severity in acute pancreatitis; CTSI = computed tomography severity index.

**Figure 3.**
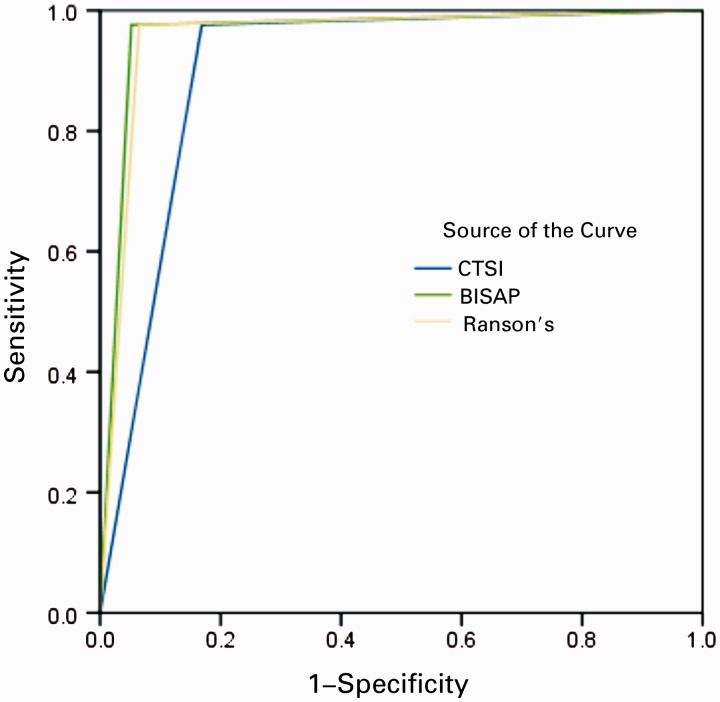
ROC curve for mortality. BISAP = bedside index for severity in acute pancreatitis; CTSI = computed tomography severity index.

ROC curves yielded an AUC of 0.962 [95% confidence interval (CI) 0.923–1.002] for BISAP in predicting SAP, 0.934 (95% CI 0.878–0.989) in predicting the development of PNec, and 0.846 (95% CI 0.772–0.920) in predicting mortality (Figure 1). Ranson’s score showed a slightly lower accuracy in predicting SAP 0.956 (95% CI 0.914–0.998) and mortality 0.841 (95% CI 0.765–0.917) (Figure 2). CTSI, as expected, was the most accurate in predicting PNec, as evidence of PNec is based on CT scan readings, with an AUC of 0.958 (95% CI 0.919–0.996) (Figure 3). AUCs for each scoring system in predicting SAP, PNec, and mortality are summarized in Table 3.

**Table 3.** Comparison of different scoring systems in predicting SAP, PNec, and mortality

Scoring systems	AUC (95% CI)		
	SAP	PNec	Mortality
BISAP score	0.962 (0.923–1.002)	0.934 (0.878–0.989)	0.846 (0.772–0.920)
Ranson’s score	0.956 (0.914–0.998)	0.927 (0.890–0.984)	0.841 (0.765–0.917)
CTSI	0.904 (0.846–0.961)	0.958 (0.919–0.996)	0.804 (0.717–0.891)

AUC = area under the curve; BISAP = bedside index for severity in acute pancreatitis; CI = confidential interval; CTSI = computed tomography severity index; PNec: pancreatic necrosis; SAP = severe acute pancreatitis

On the basis of the highest sensitivity and specificity values generated from the ROC curves, the following cut-offs were selected for further analysis: BISAP score ≥3, Ranson’s score ≥3 and CTSI ≥3. There were 45 patients with a BISAP score of ≥3, 46 with a Ranson’s score ≥3, and 54 with CTSI ≥3. Sensitivities, specificities, PPVs and NPVs of the three scoring systems in predicting SAP, PNec, and mortality are shown in Table 4.

**Table 4.** Diagnostic value of different scoring systems in predicting disease severity, PNec, and mortality

	Sensitivity (%, 95% CI)	Specificity (%, 95% CI)	PPV (%, 95% CI)	NPV (%, 95% CI)
SAP				
BISAP score	97.6 (87.4–99.6)	94.8 (87.2–98.5)	91.1 (78.7–97.5)	98.6 (92.7–99.8)
Ranson’s score	97.6 (87.4–99.6)	93.5 (85.4–97.8)	89.1 (76.4–96.3)	98.6 (92.5–99.8)
CTSI	97.6 (87.3–99.5)	83.1 (72.9–90.7)	75.9 (62.4–86.5)	98.5 (91.6–99.7)
PNec				
BISAP score	89.4 (76.9–96.4)	95.8(88.3–99.1)	93.3(81.7–98.3)	93.2(84.9–97.7)
Ranson’s score	89.4 (76.9–96.4)	94.4(86.4–98.4)	91.3 (79.2–97.5)	93.2(84.7–97.7)
CTSI	100 (92.4–100)	87.0(75.1–94.6)	90.3(81.0–96.0)	100 (94.4–100)
Mortality				
BISAP score	100 (73.4–100)	69.2 (59.5–77.7)	26.7 (14.6–41.9)	100 (95.1–100)
Ranson’s score	100 (73.4–100)	68.2 (58.5–76.9)	26.1 (14.3–41.1)	100 (95.0–100)
CTSI	100 (73.4–100)	60.7 (50.8–70.1)	22.2 (12.1–35.6)	100 (94.4–100)

BISAP = bedside index for severity in acute pancreatitis; CI = confidence interval; CTSI = computed tomography severity index; PNec = pancreatic necrosis; SAP = severe acute pancreatitis

## Discussion

Early evaluation of the severity of acute pancreatitis is essential, to allow the clinician to predict the patient’s clinical course, estimate prognosis, and determine the need for admission to the intensive care unit. Severe pancreatitis can be defined by various systems that predict complications and mortality, or by the development of the complication itself; thus, there is a difference between a predictive system that suggests complications may develop and the actual development of a complication. Several scoring systems have been developed to assist the clinician in the assessment of the severity of acute pancreatitis. The most commonly used are the Ranson’s score, the modified Glasgow scoring system, APACHE-II, CTSI and a new scoring system called BISAP [1, 2, 6–10].

With an in-hospital mortality rate of 10.1% (*n = *12), our study does not lie within the accepted range of mortality for acute pancreatitis. Singh *et al.* reported 14 (3.5%) deaths among 397 cases [2] but, in our study, 82.4% (*n = *98) of cases had been transferred from other hospitals, in contrast to only 16% transferred cases in their study. This might be the cause of higher mortality; also, as our institute lies in an underdeveloped part of India, lack of health awareness and low levels of education might be a further cause. In this part of the country, lack of proper diagnostic tests at Primary Health Centres (PHCs)—such as ultrasonography and other blood tests—leads to delayed diagnosis and referral. Moreover lack of adequate infrastructure, such as proper roads and fully equipped ambulances, are also a reason for patients presenting to us with complications.

Keeping in mind the socio-demographic differences and status of medical care in our country, we wanted to evaluate the utility of the BISAP scoring system—which has been proved to be a good predictive system for Western context—in an Indian population. Its advantage is the relative ease with which data can be acquired; this is more favourable than with the Ranson’s score, which requires data collected at admission and during the first 48 hours.

In the present study, 42 patients (35.2%) were classified as having SAP, 47 (39.5%) developed PNec, and 12 (10.1%) died. When the χ^2^ test was used to compare SAP, PNec and mortality rates, there was no significant statistical difference (*P > *0.05), indicating that BISAP accurately estimates the outcomes. It also yielded AUC values exceeding 0.9 for SAP and PNec. The three surgical risk scoring systems employed in the present study all showed comparable AUC values in predicting in-hospital mortality. Of the 12 patients who died, seven had a BISAP score of 4, and 5 had a score of 5. As expected, CTSI had 100% sensitivity in predicting PNec, while BISAP achieved 89.4%.

Other studies comparing BISAP score with other scoring systems had obtained results similar to our findings. In 2010, Papachristou *et al.* studied 185 patients with acute pancreatitis (mean age 51.7 years; 51% males), of whom 73% underwent CECT scan [3]. Forty patients (22%) developed organ failure and were classified as having SAP. Thirty-six (19%) developed PNec, and 7 (3.8%) died. AUCs for BISAP, Ranson's, APACHE-II, and CTSI in predicting SAP were 0.81, 0.94, 0.78, and 0.84, respectively [3]. In another study by Gompertz *et al.*, a BISAP score >3 had a sensitivity, specificity, PPV and NPV of 71.4%, 99.1%, 83.3% and 98.3%, respectively [10]. In 2013, Cho *et al.* also confirmed the utility of BISAP after studying 299 consecutive patients, among whom 22 (7.4%) were classified as having SAP, and 8 (2.7%) died [11]. There were statistically significant trends for increasing severity (*P < *0.001) and mortality (*P < *0.001) with increasing BISAP. The AUC for severity predicted by BISAP was 0.762 (95% CI 0.631–0.893) and by Ranson’s score was 0.804 (0.717–0.892). The AUC for mortality predicted by BISAP was 0.940 (0.863–1.018) and by Ranson’s score was 0.861 (0.734–0.988) [12].

We confirm that the BISAP score is an accurate means of risk stratification in patients with acute pancreatitis in an Indian population; the contributing data are clinically relevant and easy to obtain; the prognostic accuracy of BISAP is similar to those of the other scoring systems. Patients with a BISAP score equal to or greater than 4 invariably develop SAP or PNec and have high mortality. Hence these patients require close monitoring and intensive care.

*Conflict of interest statement:* none declared.
